# One genome’s junk is another’s garbage

**DOI:** 10.7554/eLife.23447

**Published:** 2016-12-23

**Authors:** Lydia J Bright, Douglas L Chalker

**Affiliations:** 1 Department of Biology, State University of New York at New Paltz, New Paltz, United Statesbrightl@newpaltz.edu; 2 Biology Department, Washington University in St. Louis, St. Louis, United Statesdchalker@wustl.edu

**Keywords:** *Tetrahymena thermophila*, chromosome breakage, internal eliminated sequence, genetic rearrangement, transposable element, centromere, Other

## Abstract

Experiments on a single-celled ciliate reveal how mobile genetic elements can shape a genome, even one which is not transcriptionally active.

**Related research article** Hamilton EP, Kapusta A, Huvos PE, Bidwell SL, Zafar N, Tang H, Hadjithomas M, Krishnakumar V, Badger JH, Caler EV, Russ C, Zeng Q, Fan L, Levin JZ, Shea T, Young SK, Hegarty R, Daza R, Gujja S, Wortman JR, Birren BW, Nusbaum C, Thomas J, Carey CM, Pritham EJ, Feschotte C, Noto T, Mochizuki K, Papazyan R, Taverna SD, Dear PH, Cassidy-Hanley DM, Xiong J, Miao W, Orias E, Coyne RS. 2016. Structure of the germline genome of *Tetrahymena thermophila* and relationship to the massively rearranged somatic genome. *eLife* **5**:e19090. doi: 10.7554/eLife.19090

Most of us store all kinds of junk in our garages and basements because we think that it might be useful at some time in the future. One day, we convince ourselves, we will dig out those old cleats or need that widget. Then the time comes for a good spring cleaning, and we declare that much of the junk we’ve been saving is just garbage and put it out on the curb for pick-up.

Much like basements, genomes are full of junk (such as repetitive sequences of DNA that have no obvious function). Of course, genomes also encode the instructions for making essential mRNA molecules and proteins, and these instructions need to be passed on to future generations. Many eukaryotes separate out these activities. For example, multicellular plants and animals use different cell types: the main “somatic” cells of the body express genes, while germline cells (such as egg and sperm cells) propagate DNA to offspring. Single-celled eukaryotes called ciliates, on the other hand, keep their germline genome in a germline micronucleus and their somatic genome in a separate somatic macronucleus. Now, in eLife, Robert Coyne of the J. Craig Venter Institute and colleagues – including Eileen Hamilton and Aurélie Kapusta as joint first authors – report that they have sequenced the germline genome of a ciliate called *Tetrahymena thermophila* (Hamilton et al., 2016).

When ciliates mate, their germline nuclei fuse to form a new nucleus that develops into both the somatic and germline nuclei of the offspring (Figure 1). The new germline genome remains intact and is transcriptionally inactive. To form the somatic genome, germline chromosomes break into fragments to form the chromosomes that end up in the somatic macronucleus.

**Figure 1.**
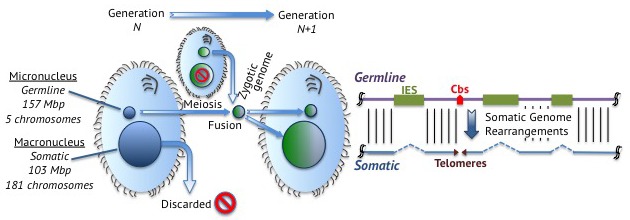
Junk DNA in *Tetrahymena*. Left: *Tetrahymena* is a single-celled ciliate that stores its germline genome in a micronucleus and its somatic genome in a macronucleus. During reproduction, two micronuclei fuse to form a zygotic nucleus that splits to form a new germline micronucleus and a new somatic macronucleus. This means that the genetic material in the somatic macronuclei of the parents is discarded. Right: Germline DNA (purple; top) remains intact in the germline micronucleus, but is processed in the somatic macronucleus to form somatic DNA (blue; bottom). Junk DNA in the form of internally eliminated sequences (IES; green boxes) is removed and the DNA fragments at chromosome breakage sites (Cbs; red arrowhead) to form five chromosomes, which are stabilized by the addition of telomeres (purple triangles) at their ends.

During this developmental process, ciliates treat junk DNA like garbage, tossing it from their somatic genomes. One can therefore identify what these cells consider to be junk by comparing the contents of their germline and somatic genomes: junk DNA is only found in the germline. The fact that ciliates keep junk DNA in their germline, even though they have developed mechanisms to remove it, may indicate that this DNA serves (or has served) some role in the lifestyle of ciliates. On the other hand, the evolutionary cost of developing or deploying mechanisms to remove the nonfunctional DNA may exceed the cost of propagating it, leading to its retention.

When Hamilton et al. compared the germline genome of *Tetrahymena* with the somatic genome, which was sequenced a decade ago (Eisen et al., 2006), they found that one third of the germline genome is discarded to form the somatic genome. In particular, they discovered that approximately 12,000 junk DNA loci are “internally eliminated sequences” that are removed during development (Figure 1), and they were able to map the exact locations of nearly 7500 of these.

The sequence and distribution of these internally eliminated sequences in the germline genome reveal a history spent combating mobile DNA elements called transposons. Most of the internally eliminated sequences appear to be descendants of transposons and were highly enriched near the center of the five metacentric chromosomes. (A metacentric chromosome has its centromere – the structure that holds sister chromatids together – at its center, whereas an acentric chromosome lacks a centromere). Some of these sequences must act as centromeric DNA, only to be removed when the acentric somatic chromosomes form.

The centromeres of most eukaryotes are rich in repetitive DNA that is packaged as heterochromatin to suppress the activity of the genes it contains. However, the germline chromosomes in *Tetrahymena* lack the form of heterochromatin found in the outer layer of the centromeres of most eukaryotes (Taverna et al., 2002). Perhaps junk DNA accumulates around centromeres in order to keep those parts of the genome that are transcribed away from the centromere, where chromatin suppresses the transcription of DNA (Fukagawa and Earnshaw, 2014).

*Tetrahymena* still forms heterochromatin to combat transposon proliferation, but not in the germline genome. The ciliates have adapted a process by which small RNA molecules direct the formation of heterochromatin to silence, then eliminate, transposons before they reach the expressed genome (Taverna et al., 2002, Mochizuki et al., 2002). To go from silencing to elimination, *Tetrahymena* cells have domesticated Tpb2p – an enzyme that normally helps transposons to hop around the genome – to cut out any DNA that is packaged in newly formed heterochromatin (Cheng et al., 2010).

A related ciliate called *Paramecium tetraurelia* removes internally eliminated sequences using a more precise method than *Tetrahymena* (Bétermier, 2004). Hamilton et al. show that all but a very small number of internally eliminated sequences in *Tetrahymena* are located within non-coding sequences, whereas thousands are located within coding sequences in *Paramecium* (Arnaiz et al., 2012). *Tetrahymena*’s imprecise excision mechanism likely prevents internally eliminated sequences (or, more accurately, their ancestral active transposons) from accumulating in protein-coding genes, which errant excision events might render non-functional.

Does any of the eliminated junk DNA contain useful stuff? There is a lot left to explore. When Hamilton et al. – who are based at institutes in the United States, Austria, the United Kingdom and China – mapped the 225 sites at which germline chromosomes break when creating the ends of the acentric somatic chromosomes, they discovered that 33 chromosome segments are not maintained in the somatic macronucleus. This appears to be more than happenstance. The sequence and position of each chromosome breakage site was conserved across *Tetrahymena* species, and new fragmentation sites appeared to be created by duplicating existing ones.

These 33 fragments encode 47 predicted open reading frames, some of which are transcribed during development before they are eliminated. Hamilton et al. propose that these fragments present a strategy for regulating gene expression during development. This is not without precedent, as the region encoding a subunit of the telomerase enzyme that is only required during development is also eliminated from the somatic genome of the ciliate *Euplotes crassus* (Karamysheva et al., 2003). In other words, the existence of junk and the means to remove it during development have been repeatedly co-opted to regulate gene expression, much like that widget in the basement proving to be useful after all. How much more of this junk DNA is more than garbage?
